# From motion to emotion: exploring challenging behaviors in autism spectrum disorder through analysis of wearable physiology and movement

**DOI:** 10.1088/1361-6579/ada51b

**Published:** 2025-01-29

**Authors:** Ali Bahrami Rad, Tania Villavicencio, Yashar Kiarashi, Conor Anderson, Jenny Foster, Hyeokhyen Kwon, Theresa Hamlin, Johanna Lantz, Gari D Clifford

**Affiliations:** 1 Department of Biomedical Informatics, Emory University, Atlanta, GA, United States of America; 2 The Center for Discovery (TCFD), Harris, NY, United States of America; 3 Department of Biomedical Engineering, Georgia Institute of Technology, Atlanta, GA, United States of America

**Keywords:** autism spectrum disorder, problem behaviors, self-injurious behavior, wearables, electrodermal activity, skin temperature, body movement

## Abstract

*Objective.* This study aims to evaluate the efficacy of wearable physiology and movement sensors in identifying a spectrum of challenging behaviors, including self-injurious behavior, in children and teenagers with autism spectrum disorder (ASD) in real-world settings. *Approach.* We utilized a long-short-term memory network with features derived using the wavelet scatter transform to analyze physiological biosignals, including electrodermal activity and skin temperature, alongside three-dimensional movement data captured via accelerometers. The study was conducted in naturalistic environments, focusing on participants’ daily activities. *Main results.* Our findings indicate that the best performance in detecting challenging behaviors was achieved using movement data. The results showed a sensitivity of 0.62, specificity of 0.71, F1-score of 0.36, and an area under the ROC curve of 0.71. These results are particularly significant given the study’s focus on real-world scenarios and the limited existing research in this area. *Significance.* This study demonstrates that using wearable technology to record physiological and movement signals can detect challenging behaviors in children with ASD in real-world settings. This methodology has the potential to greatly improve the management of these behaviors, thereby enhancing the quality of life for children with ASD and their caregivers. This approach marks a significant step forward in applying the outcome of ASD research in practical, everyday environments.

## 1. Introduction

Autism spectrum disorder (ASD) is a neurodevelopmental disorder characterized by persistent challenges in social interaction, communication, and restricted and repetitive patterns of behavior, interests, or activities (American Psychiatric Association 2022). These symptoms typically manifest in early childhood and can cause significant impairments in various aspects of daily functioning. ASD is a heterogeneous disorder, with individuals presenting a wide range of symptom severity and intellectual functioning (Jeste and Geschwind 2014, Ameis 2017, Masi *et al* 2017). The exact etiology of ASD remains unknown, but research suggests that a complex interplay of genetic, environmental, and developmental factors contributes to its development (Shaw *et al* 2014, Bölte *et al* 2019, Taylor *et al* 2020, Yoon *et al* 2020).

Individuals with ASD may exhibit a diverse array of challenging behaviors that significantly impact their daily functioning and quality of life (Lindor *et al* 2019, Edelson 2022). These behaviors encompass a broad spectrum of actions that can prove challenging for caregivers and professionals to manage effectively. Notable examples include repetitive behaviors, restricted interests, sensory sensitivity, social communication challenges, emotional regulation difficulties, aggression, self-injury, elopement, non-compliance, sleep problems, and eating challenges. Each of these behaviors presents unique challenges, necessitating the development and implementation of tailored interventions to mitigate their impact on the individual’s life and facilitate their successful integration into community and educational settings.

Among the various categories of challenging behaviors associated with ASD, self-injurious behavior (SIB) is particularly distressing (Minshawi *et al* 2014, Edelson 2016, Soke *et al* 2016). SIB involves actions inflicted by an individual upon themselves, resulting in physical harm (American Psychiatric Association 2022). Examples of SIB include but are not limited to, head banging against hard surfaces, self-biting, skin scratching or cutting, self-hitting with objects or body parts, and hair pulling (Tate and Baroff 1966). These behaviors can lead to significant physical injury and often serve as indicators of underlying distress or unmet needs.

The etiology of SIB in ASD is multifaceted, encompassing factors such as sensory dysregulation, communication deficits, frustration, anxiety, and, in some cases, medical issues that the individual has difficulty articulating (Carr 1977, Guess and Carr 1991, Kurtz *et al* 2003, 2012, Symons 2011, Devine 2014). Addressing SIB in ASD requires a comprehensive, multidisciplinary approach that integrates behavioral interventions, environmental modifications, sensory integration strategies, enhanced communication supports, medications, treatment of underlying medical conditions and even electroconvulsive therapy (ECT) (Smith *et al* 2005, Devlin *et al* 2009, Chezan *et al* 2017, D’Agati *et al* 2017, Erturk *et al* 2018, Sabus *et al* 2019, Alakhzami and Chitiyo 2022). This holistic approach aims to mitigate the underlying causes of SIB, promoting safer behavior patterns and improving overall well-being.

The immediate identification, or ideally, the prediction of SIB and other problem behaviors in individuals with ASD, stands as a crucial objective for contemporary research and clinical practice. With prevalence rates estimated at 42% (Steenfeldt-Kristensen *et al* 2020), SIB not only significantly deteriorates the quality of life for individuals with ASD but also imposes profound challenges on families, healthcare systems, and societal resources (Lecavalier *et al* 2006, Ianni *et al* 2015, McGuire *et al* 2016, Shawler *et al* 2023). Beyond the considerable economic burdens stemming from direct medical expenses, specialized care, and lost caregiver productivity, the human cost of delayed intervention in managing SIB can be profound.

The significance of instantaneous detection or predictive anticipation of SIB cannot be overstated. By identifying or predicting the onset of such behaviors in real-time, it becomes possible to intervene immediately, preventing potential harm to the subjects before it occurs. This level of prompt intervention can dramatically reduce the physical risks associated with SIB, such as injuries or more severe health complications that might arise from delayed response.

Applications of real-time identification and predictive analytics in SIB could revolutionize how care is provided for individuals with ASD. Wearable technology that monitors physiological signals and movement patterns could serve as an early warning system, signaling caregivers and healthcare professionals to imminent SIB episodes. Such technology could employ machine learning algorithms trained to recognize precursors to SIB, enabling preemptive action to avert the behavior.

Moreover, integrating this technology in educational and therapeutic settings could promote safer environments for individuals with ASD, ensuring immediate response to their needs and minimizing disruptions to their learning and social integration. In clinical contexts, real-time monitoring could inform more personalized and dynamic treatment plans, adjusting interventions as the individual’s state changes throughout the day.

Ultimately, the applications of immediate SIB identification and predictive technology extend beyond preventing physical harm; they promise to enhance the overall well-being of individuals with ASD, easing the emotional and logistical strains on families and caregivers. Such advancements herald a future where individuals with ASD receive more proactive, personalized care, significantly improving their quality of life and integration into society.

## 2. Problem specification

The primary objective of this study is to evaluate the efficacy of wearable physiology and movement sensors in identifying a spectrum of challenging behaviors, including SIB, in children and teenagers with ASD. This investigation aims to leverage the capabilities of wearable technology (Simm *et al* 2016, Koo *et al* 2018, Taj-Eldin *et al* 2018, Black *et al* 2020, Kientz *et al* 2020, Francese and Yang 2022, Cano *et al* 2023) to establish a non-invasive, early detection and monitoring system for these behaviors. While the study’s focus is not on developing intervention strategies, the rapid and accurate identification of such behaviors may lay the groundwork for future enhancements in timely and effective intervention methods.

This study explores approaches for multimodal analysis, integrating wearable sensors to collect physiological and movement data essential for identifying problem behaviors and SIB. Specifically, the research utilizes accelerometers for movement analysis, electrodermal activity (EDA) sensors for measuring emotional arousal, and skin temperature sensors to gather relevant data. It is important to note that the scope of wearable technology is not limited to the sensors selected for this study. Other devices, such as heart rate variability monitors and gyroscopes, could potentially provide additional insights into the complex physiological and movement patterns associated with problem behaviors in children and teenagers with ASD (Fioriello *et al* 2020, Alban *et al* 2023, Cano *et al* 2023, Farkas *et al* 2023).

This research distinguishes itself from previous efforts by conducting the study in real-world settings or naturalistic observation rather than controlled laboratory environments. Data collection occurs as participants, all children and teenagers, engage in their usual daily activities, thus ensuring that the findings reflect genuine behavior manifestations in natural settings. Over the course of several years, this approach allows for the observation of participants’ behaviors and physiological responses in a variety of contexts, enhancing the ecological validity of the data.

Unlike prior studies that may focus on a single type of problem behavior, this work aims to identify a broad range of problem behaviors, including but not limited to SIB, aggression, and other disruptive behaviors. This expansive focus is vital for developing a comprehensive understanding of the challenges faced by individuals with ASD and for informing the development of more nuanced intervention strategies.

The participant group comprises children and teenagers with ASD, with data recorded over a couple of years to capture the developmental and behavioral changes that occur within this timeframe. Recruitment and data collection are conducted with the utmost adherence to ethical standards, prioritizing informed consent, privacy, and the safety of all participants. Through this longitudinal approach, the study endeavors to create a robust and versatile system for the early detection of problem behaviors in ASD, utilizing the latest in wearable technology and data analytics. This innovative tool is expected to significantly contribute to the field, offering caregivers, healthcare professionals, and researchers invaluable resources for monitoring, understanding, and ultimately improving the quality of life for children and teenagers with ASD.

## 3. Study design

### 3.1. Decoding motion and emotion: sensor modalities in focus

In the quest to understand the nuanced expressions of ASD, this study harnesses a blend of wearable technology, complemented by video recordings, to capture a comprehensive dataset of physiological and movement indicators. The wearable sensors employed include EDA, accelerometers, and skin temperature sensors, each offering a unique window into the subtle shifts in emotion and motion that characterize the daily experiences of individuals with ASD. The video recordings are used in the manual labeling process, where observers assign labels based on observed behaviors. In addition, they are setting a foundation for future studies where automated video analysis might be employed.

EDA, also known as skin conductance, plays a pivotal role in measuring the skin’s electrical conductance, which varies with moisture levels. EDA provides insight into the autonomic nervous system’s reactions, highlighting emotional arousal or stress. By distinguishing between phasic (immediate) and tonic (sustained) responses, we delve deeper into the temporal dynamics of stress and arousal, which is essential for pinpointing the onset of behaviors. This measure is especially pertinent for individuals with ASD, who may struggle to communicate their emotional states verbally. Thus, EDA serves as a non-verbal indicator of emotional and stress responses.

Accelerometers contribute to this multimodal analysis by capturing detailed three-dimensional movement across the *X*-axis (horizontal), *Y*-axis (vertical), and *Z*-axis (depth). Such detailed movement tracking is instrumental in identifying patterns of behavior specific to ASD, such as repetitive or agitated motions. Recognizing these patterns enables a proactive approach to managing problem behaviors, potentially mitigating their occurrence through timely intervention.

Skin Temperature monitoring adds another dimension to our understanding by reflecting physiological state changes. Variations in skin temperature can indicate emotional stress or discomfort (Vinkers *et al* 2013), enriching the dataset with another parameter that correlates with emotional states.

Integral to this multimodal approach is the use of cameras and video recording. These tools are pivotal for the annotation process, providing the necessary behavioral context to the physiological and movement data captured by the wearables. Videos serve as a key reference for accurately labeling the recorded data, facilitating a nuanced analysis of the precursors and manifestations of problem behaviors in ASD. While the current study focuses on wearable-derived data, future research will aim to merge wearable and video analysis, as well as our sleep (Kiarashi *et al* 2024b) and digital health record analysis (Kiarashi *et al* 2025), for a more comprehensive behavioral understanding and prediction.

This ensemble of sensors, digital health records, and video recording tools offers a multidimensional view of the ASD experience, combining the physiological and physical movement markers with behavioral context. This holistic approach not only aims to decode the complex interplay between motion and emotion in ASD but also sets the stage for future innovations in monitoring, understanding, and intervening in the spectrum of behaviors associated with ASD.

### 3.2. Data collection and labeling process

The study was conducted at The Center for Discovery (TCFD) in New York State, which is recognized for its residential programs tailored to individuals with multiple disabilities and autism. Eleven male students, aged between 10 and 20 years, who resided at the CFD, participated in the study (see table 1). The Institutional Review Board at the CFD provided approval, and informed consent was obtained from the parents or guardians of each participant.

**Table 1.** Dataset which is used in this study.

Subject ID	Age start	Age end	Sex	# of sessions	Modalities	Total duration of labeled sessions (h)	Total duration of problem behaviors (h)	Prevalence of problem behaviors (%)
S01	17	20	M	23	EDA, ACC, Tsk	21.4	2.7	13
S03	14	15	M	N/A	—	N/A	N/A	N/A
S06	14	16	M	6	EDA, ACC, Tsk	5.1	0.1	3
S07	10	13	M	23	EDA, ACC, Tsk	14.6	1.4	10
S08	16	17	M	12	EDA, ACC, Tsk	12.8	0.5	4
S09	12	14	M	24	EDA, ACC, Tsk	20.3	2.1	10
S10	14	15	M	8	EDA, ACC, Tsk	8.1	1.1	14
S11	14	15	M	22	EDA, ACC, Tsk	12.8	0.3	2
S12	20	21	M	12	EDA, ACC, Tsk	10.8	4.6	43
S13	19	20	M	6	EDA, ACC, Tsk	4.8	2.2	46
S21	14	15	M	33	EDA, ACC, Tsk	19.3	2.6	13

Behavior monitoring was systematically undertaken by staff members from the Psychology department, who carefully defined and identified specific behaviors of interest for each participant. To capture these behaviors within their natural context, video recordings of classroom activities were made during typical school days, utilizing a multi-camera system designed to be as unobtrusive as possible. The length of these video recordings averaged 25 min.

The task of annotating these videos was carried out using Noldus The Observer XT 15 software by a trained observer. This process involved marking each identified behavior’s onset and offset times, with latency times between behaviors ranging from five to ten seconds, depending on the operational definitions established for each participant. The annotated behaviors were then exported into CSV files for further analysis.

Concurrently, EDA data were collected using Q-Sensor wristbands (Affectiva Inc. Cambridge, MA), which recorded data at a sampling rate of 32 Hz. These wristbands, fitted with Ag/AgCl dry electrodes, were worn by participants on either the wrist or ankle (van Dooren *et al* 2012), selected based on the individual’s comfort and tolerance. Notably, ankle placement was chosen for those students who experienced issues with wrist placement, ensuring consistent data collection across all participants. The EDA data collected were downloaded into CSV files for analysis using Affectiva’s proprietary software.

The criteria for video selection were stringent, focusing only on instances where a participant exhibited a behavior of interest while also wearing a Q-sensor and recorded a valid EDA signal. A trained professional visually inspected EDA signals to identify any potential artifacts that could compromise data quality. All data collection occurred in a temperature-controlled classroom to maintain a consistent environment.

Significantly, the total duration of physiological and movement data collected surpasses that of the video recordings, indicating that a considerable portion of the data remains unlabeled. This discrepancy highlights the challenge of having a large volume of unlabeled data, underscoring the need for future studies to bridge this gap and enhance the labeling process for a more comprehensive analysis.

Focused on fifteen individually operationalized challenging behaviors, the study targets a broad spectrum of actions, including (1) out-of-seat, (2) elopement, (3) motor stereotypies, (4) aggression, (5) SIB, (6) self-injurious jump, (7) hand bite, (8) hand(s) on ears, (9) dropping, (10) jumping, (11) disruptive behavior, (12) chin pressure, (13) head tapping, (14) agitation, and (15) slap.

### 3.3. Feature engineering

The intricate analysis of physiological signals, such as EDA, heart rate, blood pressure, respiratory rate, pupil dilation, skin temperature, and muscle activity, plays a critical role in deciphering the human body’s response to stress and arousal (Shu *et al* 2018, Ghiasi *et al* 2020, Van Der Mee *et al* 2021). During acutely stressful or arousing situations, activation of the sympathetic nervous system (SNS) stimulates the eccrine sweat glands, which affects the electrical properties of the skin, leading to changes in EDA (Critchley 2002, Boucsein 2012, Svetlak *et al* 2013). Phasic EDA, or skin conductance responses, reflects rapid, short-term changes in skin electrical resistance within a few seconds, representing transient electrodermal responses to specific stimuli or events. Tonic EDA, or skin conductance level (SCL), represents the relatively longer-lasting, baseline level of skin conductance over a specific time period, reflecting general arousal or stress levels (Daviaux *et al* 2020, Greco *et al* 2021).

During acute stress induction paradigms, the following EDA changes are typically observed: there is an increased SCL, indicating that exposure to the stressor is associated with heightened sympathetic arousal and stress response. After the termination of the stressor, the SCL gradually decreases within a few minutes, reflecting the recovery from the acute stress response as the sympathetic activation subsides. Therefore, the tonic SCL component of EDA provides a reliable measure of the body’s physiological stress response during acute stress induction, with an initial increase followed by a gradual decrease after the stressor is removed.

Emerging from spectral analysis, a specific frequency band (0.045–0.25 Hz) has been recognized for its heightened sensitivity to central sympathetic control in states of rest (Posada-Quintero *et al* 2016a, 2016b). Nonetheless, this frequency domain remains less examined in scenarios involving physical exertion, where a shift in frequencies corresponding to autonomic dynamics is anticipated as exercise intensifies. Research indicates that the upper-frequency limit (*F*_max_) enveloping EDA spectral power shifts markedly to higher frequencies amidst prolonged low-intensity ($F_{\textrm{max}}\approx 0.28$ Hz) and vigorous-intensity exercise ($F_{\textrm{max}}\approx 0.37$ Hz) compared to rest (Posada-Quintero *et al* 2018).

Analyzing EDA’s phasic and tonic components, particularly across diverse levels of physical activity, poses significant challenges. These components manifest multi-scale characteristics, demanding advanced analytical approaches for their effective separation and thorough examination under various physiological conditions. The limitations of conventional methodologies in capturing the entirety of physiological signals, especially under the influence of exercise or acute stress, underscore the demand for a more sophisticated analytical framework.

The wavelet scatter transform (WST) (Mallat 2012) answers this call, offering a groundbreaking approach in signal processing. Its foremost advantage is the ability for multilayered decomposition, enabling intricate analysis across an extensive frequency spectrum. This feature empowers the WST to adeptly isolate and delve into the complex structures of physiological signals like EDA, distinguishing it as a premier tool for revealing detailed SNS activities.

Unique to the WST is its capability to transcend the conventional binary of phasic and tonic components. Instead, WST facilitates the exploration of EDA through diverse signal components derived from various wavelet bandwidths. This approach allows for a more refined understanding of the signal, accommodating the intricate dynamics of physiological responses without being confined to the traditional categorizations of tonic and phasic activities. Such granularity in analysis is crucial for capturing the subtleties of sympathetic control, especially in contexts where these dynamics exhibit significant variation, such as during physical activity or stress exposure.

WST’s versatility extends to examining other crucial signals within research, including accelerometer outputs and skin temperature changes. The multifaceted nature of these signals demands an analytical tool as robust and adaptable as WST. For accelerometer data, the WST’s nuanced dissection of movement patterns unveils both subtle and distinct behaviors, which are invaluable in ASD research, among other things. Similarly, its adeptness at parsing skin temperature variations furnishes a comprehensive perspective on physiological responses, pivotal for thorough signal analysis.

Addressing the challenge of intra-class variability, which impedes the effective discrimination between different classes, is crucial in classification tasks. WST offers a methodical approach to mitigate this issue by constructing representations that are locally invariant, stable, and informative while also preserving signal norm and the majority of inter-class variabilities. This transform operates similarly to convolutional neural networks (CNNs) by processing the input signal through a series of linear filters, pooling, and nonlinear operations (Mallat 2016). However, unlike CNNs, where filters are adaptively tuned, the scattering transform employs predefined wavelets, achieving a deep signal representation. Specifically, it cascades wavelet transform moduli with an averaging operation to ensure translation invariance and stability against deformations like time warping (Mallat 2012).

We construct a 2-layer scattering network utilizing Gabor wavelets (Mallat 2008), with the central frequencies of the mother wavelets in the first and second filter banks calculated as follows

\begin{align*} \omega_0^{\langle 1 \rangle} = \left(\frac{1+2^{-1/Q_1}}{2}\right)\times \frac{f_\textrm{s}}{2} = 13.66~\textrm{Hz},\end{align*}

\begin{align*} \omega_0^{\langle 2 \rangle} = \left(\frac{1+2^{-1/Q_2}}{2}\right)\times \frac{f_\textrm{s}}{2} = 12.00~\textrm{Hz},\end{align*}

where the quality factors *Q*_1_ = 2 and *Q*_2_ = 1 represent the number of wavelets per octave for the first and second filter banks, respectively, and $f_\textrm{s} = 32$ Hz is the sampling rate. We designed this scattering network to ensure that the resulting representation is invariant to a 5 s translation. Other wavelets $\psi_{j_k}^{\langle k \rangle}(t)$ in the filter banks are derived by dilating the mother wavelets $\psi_{0}^{\langle k \rangle}(t)$ by a factor of $2^{1/Q_k}$

\begin{equation*} \psi_{j_k}^{\langle k \rangle}\left(t\right) = 2^{-{j_k}/{Q_k}}\psi_{0}^{\langle k \rangle}\left(2^{-{j_k}/{Q_k}}t\right),\end{equation*}

where $k \in \{1, 2\}$ indicates the layer index in the scattering network and $j_k \in \{0,1,2,\ldots,J_k-1\}$ indicates the scale index. In the Fourier domain, these filter banks can be represented by

**Figure 1.**
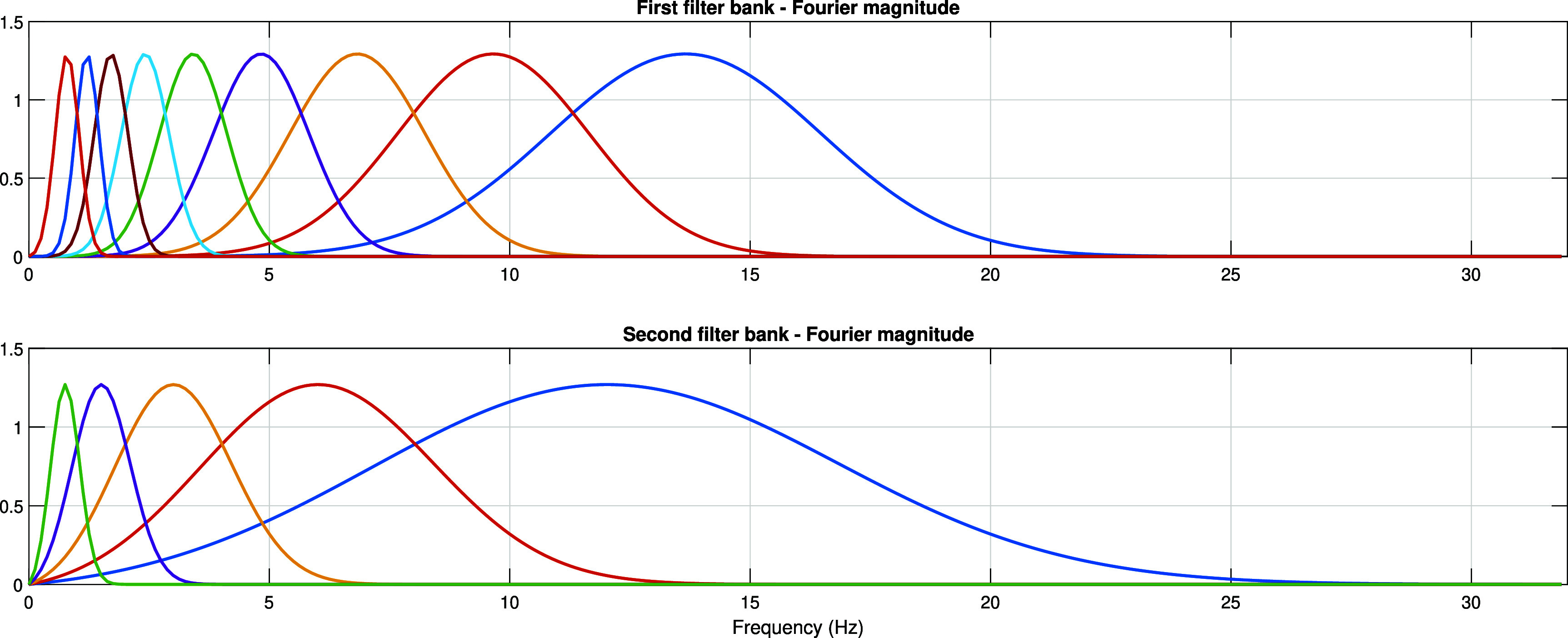
Magnitudes of the frequency spectra of the wavelets in the two filter banks.

\begin{equation*} \hat\psi_{j_k}^{\langle k \rangle}\left(\omega\right) = \hat\psi_{0}^{\langle k \rangle}\left(2^{{j_k}/{Q_k}}\omega\right),\end{equation*}

whose magnitudes are demonstrated in figure 1.

In the time domain, this 2-layer scattering network is summarized as follows

\begin{align*} S_0 x &amp; = x \star \phi\end{align*}

\begin{align*} U_1 x &amp; = |x \star \psi_{j_1}|\end{align*}

\begin{align*} S_1 x &amp; = |x \star \psi_{j_1}|\star \phi\end{align*}

\begin{align*} U_2 x &amp; = ||x \star \psi_{j_1}|\star \psi_{j_2}|\end{align*}

\begin{align*} S_2 x &amp; = ||x \star \psi_{j_1}|\star \psi_{j_2}|\star \phi,\end{align*}

where $\star$ denotes convolution and $|\cdot|$ is the complex modulus operator. In (4*a*), the zeroth-order scattering coefficient is computed by low-pass filtering the original signal *x* using the approximation function *φ*, which removes high-frequency content. This content is recovered in (4*b*) through convolution with wavelets $\psi_{j_1}$, capturing signal variations at different scales *j*_1_. The complex modulus operator $|\cdot|$ in (4*b*) does not result in significant information loss, as *x* can be reconstructed from $|x \star \psi_{j}|$ up to a global phase (Mallat and Waldspurger 2015). The primary information loss occurs during low-pass filtering, necessary for generating shift-invariant features. First-order scattering coefficients (4*c*) are obtained by low-pass filtering the first-order wavelet scattering modulus $U_1x$. The subsequent wavelet scattering modulus (4*d*) is calculated by convolving $U_1x$ with second-layer wavelets $\psi_{j_2}$. Finally, second-order scattering coefficients (4*e*) are derived. This process can be extended to higher orders, but we limit it to the second order due to the diminishing energy of higher-order coefficients, which do not significantly enhance classification results (Andén and Mallat 2014, Waldspurger 2017).

This structure parallels a CNN with convolutional layers (wavelet transforms $x \star \psi$), nonlinearities (modulus operations $|\cdot|$), and average pooling (low-pass filtering $|\cdot|\star \phi$). However, unlike CNNs, the filters are predefined rather than data-driven, and there is no weight sharing among different scales. For a detailed discussion, see Rad *et al* (2019).

In this study, we extract wavelet scattering coefficients for EDA, three accelerometer channels, and skin temperature. Due to the substantial overlap in the frequency domain of non-orthogonal Gabor wavelets (refer to figure 1), the derived scattering coefficients exhibit redundancy. To expedite the analysis and reduce memory consumption, we downsample the features by a factor of 5. Consequently, each biosignal or accelerometer channel yields 26 features, culminating in an aggregate of 130 features.

### 3.4. Classification

In the context of analyzing time-series data, the recurrent neural networks (RNN), and specifically long short-term memory (LSTM) networks (Hochreiter and Schmidhuber 1997, Gers *et al* 2000), are highly suitable. RNNs are designed to handle sequential data, offering the ability to process inputs in a way that takes into account the temporal dynamics. LSTMs, a variant of RNNs, are particularly effective due to their ability to remember information over extended time intervals. This feature is crucial for analyzing physiological data where the temporal context and the persistence of certain states, like prolonged stress or movement patterns, are important. LSTMs can capture and learn from these long-term dependencies in the data, making them an appropriate choice for classifying and predicting behavioral outcomes from the complex and temporally dependent datasets typical in ASD research.

Our focus is on detecting challenging behaviors in ASD using time-series data from EDA, accelerometer readings, and skin temperature. We employ a bidirectional LSTM (BiLSTM) network, which, unlike standard LSTM, is not a causal model. BiLSTM processes data in both forward and backward directions, providing a comprehensive view of the entire sequence, which is advantageous for detection tasks. However, for prediction tasks where future data cannot be utilized, a standard LSTM, which is causal and relies only on past and present data, would be appropriate. This distinction highlights the specific applicability of different LSTM variations in biosignal analysis.

The architecture of the proposed network consists of one layer of BiLSTM with 200 hidden units (100 for each forward and backward LSTM). It is followed by a leaky rectifier linear unit (Leaky ReLU) layer, a fully connected layer, and a softmax layer. We meticulously evaluated various combinations to determine the optimal architecture empirically. This evaluation included different numbers of BiLSTM layers, varying numbers of memory cells per layer, multiple activation functions, different numbers of fully connected layers, and various parameters for the Leaky ReLU layer. The Leaky ReLU function used in this work is

\begin{equation*} f\left(x\right) = \begin{cases} x &amp; \textrm{for}~x> 0\\ 0.5 x &amp; \textrm{for}~x\unicode{x2A7D} 0 \end{cases}.\end{equation*}

## 4. Results

This study’s exploration into detecting challenging behaviors in children with ASD was carried out using two distinct analytical frameworks: general (population-based) models and patient-specific models. Both frameworks utilized a variety of physiological and movement data modalities—namely EDA, accelerometer data, skin temperature, and a combination of all three modalities. In this section, we present the results for each individual modality as well as the combined modality and compare their performance.

### 4.1. General (population-based) models

The general model was trained using data from all participants and evaluated using 10-fold cross-validation, where the data were split into training and test sets for each fold. After completing the cross-validation, the test results from all folds were stacked to generate a final receiver operating characteristic (ROC) curve. The performance metrics, including sensitivity, specificity, positive predictive value (PPV), negative predictive value (NPV), F1-score, and area under the ROC curve (AUROC), for each modality (EDA, accelerometer, skin temperature, and a combination of all signals), are summarized in table 2.

**Table 2.** Comparison of performance metrics across different modalities in general (population-based) models evaluated using 10-fold cross-validation.

Modalities	Sensitivity	Specificity	PPV	NPV	F1-Score	AUROC
EDA	0.259	0.793	0.166	0.871	0.202	0.524
ACC (3)	0.623	0.708	0.253	0.922	0.360	0.710
Tsk	0.787	0.452	0.186	0.930	0.301	0.629
All	0.610	0.674	0.230	0.916	0.333	0.688

**Figure 2.**
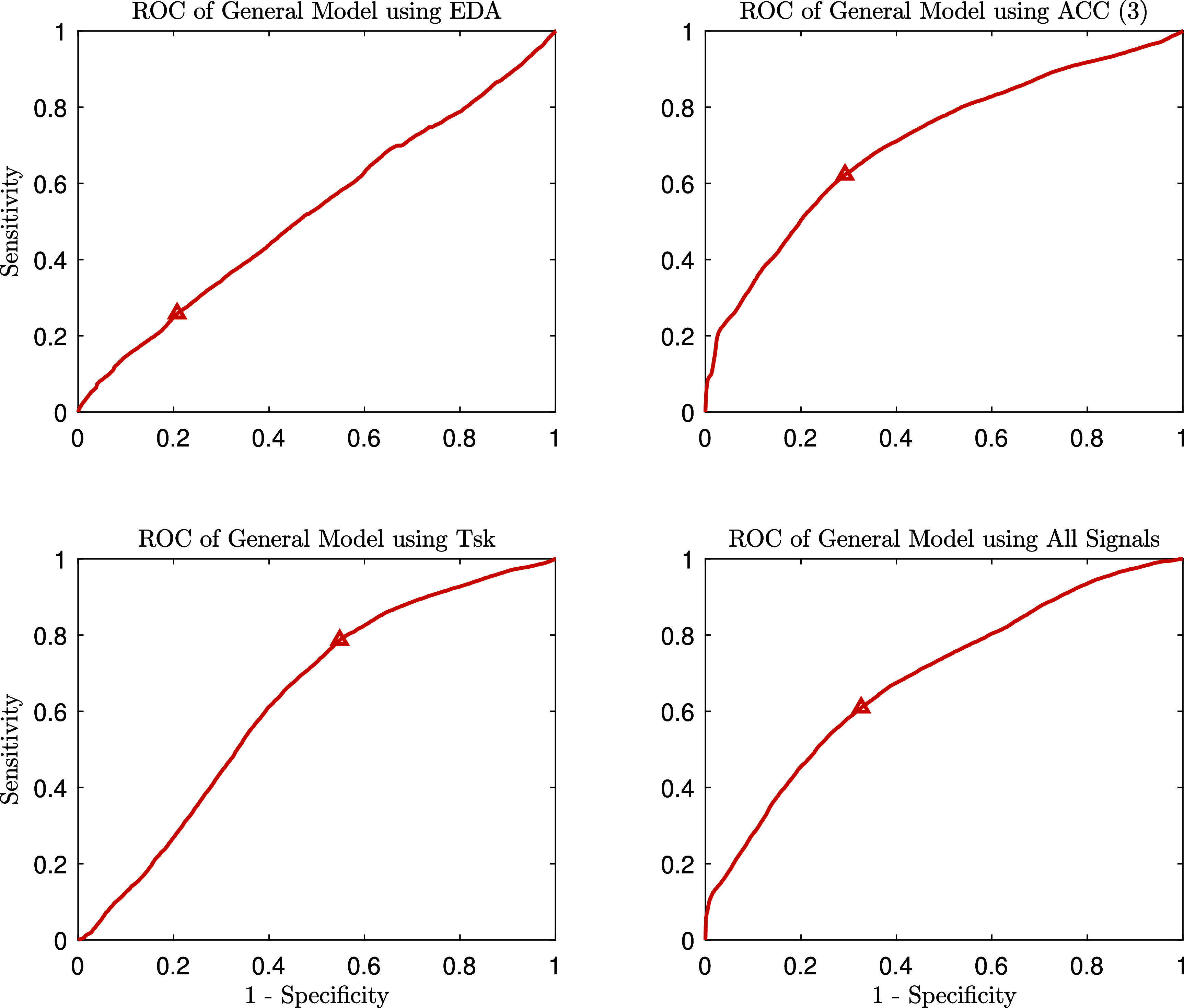
ROC curves for the general (population-based) model across different modalities (EDA, accelerometer, skin temperature, and a combination of all signals). The operating point on each ROC curve is indicated by a small triangle.

Among the different signal types, the best results were obtained using the movement data captured by the three accelerometer channels. The accelerometer data demonstrated superior performance compared to EDA and skin temperature, likely due to the high temporal resolution and multidimensional nature of accelerometer signals. These signals are particularly suited for detecting the motor patterns associated with challenging behaviors in ASD. A visual representation of the ROC curves for each modality is shown in figure 2.

### 4.2. Patient-specific models

In contrast to the general model, the patient-specific models were trained and tested using a session-wise leave-one-out framework. For each participant, the model was trained on all but one session and tested on the remaining session. This process was repeated for all sessions, allowing us to generate a cross-validated ROC curve by stacking the test results across sessions. For example, for participant S01, who had 23 sessions, the model underwent 23 cross-validation iterations, each using a different session as the test set.

**Figure 3.**
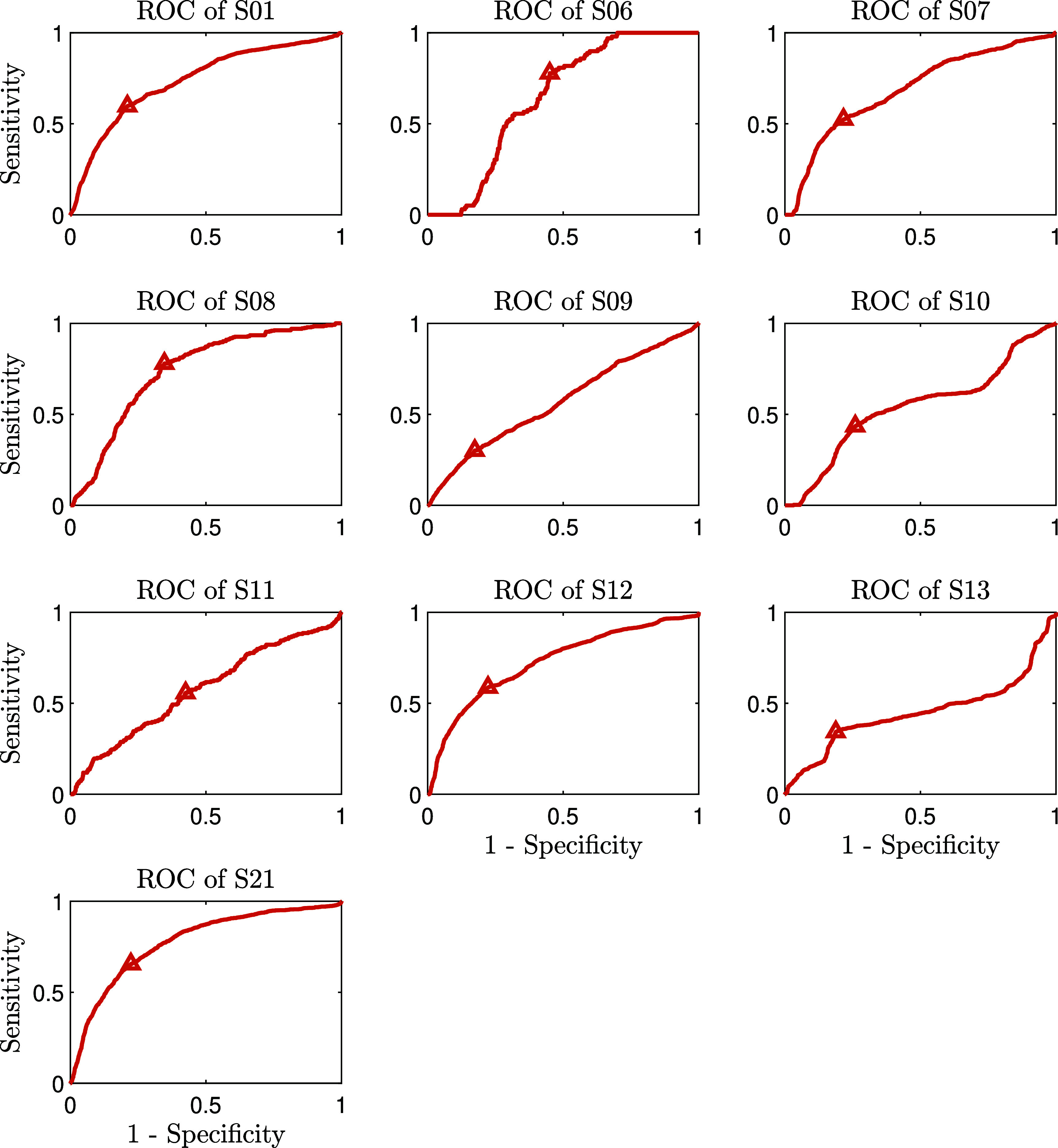
ROC curves for the patient-specific models using accelerometer data. The operating point on each ROC curve is represented by a small triangle.

Similar to the general models, we observed that the patient-specific models using accelerometer data outperformed those using other modalities, such as EDA, skin temperature, and the combination of all modalities together. This pattern reinforces the importance of movement data in detecting challenging behaviors in ASD across both modeling frameworks. The performance metrics for the patient-specific models, specifically those using accelerometer data, are summarized in table 3. Moreover, the ROC curves for each participant, generated using the session-wise procedure, are presented in figure 3. These curves illustrate the performance of accelerometer-based models across participants.

**Table 3.** Performance metrics for patient-specific (personalized) models using accelerometer data, evaluated through a session-wise leave-one-out cross-validation approach.

Subject ID	# of sessions	Modalities	Sensitivity	Specificity	PPV	NPV	F1-Score	AUROC
S01	23	ACC (3)	0.597	0.790	0.295	0.930	0.394	0.736
S06	6	ACC (3)	0.778	0.550	0.045	0.989	0.086	0.645
S07	23	ACC (3)	0.524	0.784	0.205	0.939	0.295	0.689
S08	12	ACC (3)	0.779	0.653	0.086	0.986	0.154	0.739
S09	24	ACC (3)	0.301	0.826	0.167	0.911	0.215	0.570
S10	8	ACC (3)	0.436	0.740	0.216	0.889	0.289	0.542
S11	22	ACC (3)	0.557	0.575	0.031	0.982	0.059	0.574
S12	12	ACC (3)	0.587	0.777	0.664	0.716	0.627	0.727
S13	6	ACC (3)	0.343	0.812	0.618	0.583	0.441	0.451
S21	33	ACC (3)	0.655	0.777	0.318	0.934	0.428	0.776

### 4.3. Performance comparison: general vs. patient-specific models

Contrary to our anticipations of superior performance, patient-specific models did not consistently outperform the general models. This unexpected outcome can largely be attributed to the data limitations inherent in constructing highly individualized models. The machine learning algorithms employed, characterized by their substantial data requirements, faced challenges in optimizing performance due to the scarcity of extensive, individualized datasets. This limitation highlights the critical need for robust, large-scale data to fully leverage the potential of patient-specific approaches in accurately detecting challenging behaviors.

In comparison, general models, which utilize broader patterns across a population, maintain a level of performance that, in some cases, surpasses that of the patient-specific models. This observation underscores the trade-off between the theoretically ideal scenario of personalized models and the practical realities of data availability and model training constraints.

Despite the current limitations observed with patient-specific models, we posit that this approach holds significant promise for future research, particularly with the strategic application of transfer learning techniques. Transfer learning offers a viable path forward in scenarios of limited data, allowing models to leverage pre-learned patterns from related tasks or populations to enhance performance on new, data-scarce patient-specific tasks. This methodology could bridge the gap between the data-hungry nature of deep learning algorithms and the sparse datasets typically available for individual patients, potentially unlocking the superior precision and customization of patient-specific models without the need for extensive individual data.

The findings from our comparative analysis not only shed light on the complexities of applying machine learning techniques in the ASD context but also chart a course for future investigations. Embracing transfer learning and other innovative approaches to mitigate data limitations could revolutionize the detection and management of challenging behaviors in ASD, aligning the theoretical advantages of patient-specific models with practical implementation feasibility.

## 5. Discussion

This study embarked on a comprehensive exploration to detect problem behaviors in children with ASD using a multimodal approach that integrates EDA, skin temperature, and three-dimensional accelerometer signals. Our comprehensive analysis underscores the pronounced superiority of accelerometer signals in this domain, attributing to their intrinsic sensitivity to movement, exceptional temporal resolution, and capacity to provide multidimensional insights. These characteristics are indispensable for the precise quantification of movement dynamics and crucial for recognizing the specific motor patterns often associated with problem behaviors in autistic children.

Accelerometer signals’ dominance in detecting and characterizing behavioral episodes is contrasted with the limitations observed in the performance of EDA and skin temperature as reliable indicators. The theoretical appeal of EDA, predicated on its ability to reflect SNS activity, was challenged by practical limitations. These included its susceptibility to non-specific responses influenced by a multitude of factors beyond stress or emotional arousal, such as environmental conditions and physical activities. Furthermore, the considerable inter-individual variability in EDA responses and the propensity for artifacts introduced by movement significantly compromised its utility as a predictive tool in our study context.

Similarly, skin temperature, while offering some insights into physiological states, was found inadequate for the rapid detection of problem behaviors due to its relative insensitivity to the quick onset of such events. The limitations inherent to EDA and skin temperature underscore the complexity of deploying single physiological measures for the prediction of problem behaviors in ASD, thereby highlighting the necessity for a multimodal approach. This approach should synergistically integrate various physiological indicators to construct a more robust and accurate prediction model.

While our study found limitations in the performance of EDA and skin temperature for rapid detection of problem behaviors, it is important to emphasize that these physiological measures may still hold significant value in other aspects of autism research and clinical applications. Our findings do not negate the potential utility of these measures, but rather highlight the need for careful consideration of their application context. We conjecture that EDA and skin temperature data may prove particularly useful in applications requiring lower temporal resolution or longer processing times. For instance, these measures could potentially contribute to assessing the severity or frequency of problem behaviors over extended periods, or in tracking long-term patterns of physiological arousal in individuals with ASD. Such applications might provide valuable insights into the overall stress levels, emotional regulation capacities, or autonomic nervous system functioning of individuals with ASD. Further research is needed to explore these potential uses and to determine how EDA and skin temperature data can be most effectively integrated into comprehensive assessment and monitoring strategies for ASD.

In contrast to previous research conducted predominantly in controlled laboratory settings, our study ventures into the complexity of real-world environments. This approach addresses a critical gap in the literature by evaluating the applicability of wearable technology and innovative algorithms in everyday scenarios. The transition from controlled environments to real-world conditions introduces several challenges, notably the variability and unpredictability of external factors that can influence physiological signals and movement data. These factors range from environmental conditions affecting skin temperature and EDA to the diverse range of activities and interactions children may engage in outside of a laboratory setting.

Moreover, the study confronts the prevalent issue of class imbalance in machine learning algorithms designed for detecting SIB and other challenging behaviors in ASD. Problematic behaviors, including SIB, are inherently rare events, leading to a significant class imbalance that poses challenges for algorithm design and performance evaluation. This imbalance can skew the predictive models, making them overly sensitive to the majority class and potentially underperforming in reliably identifying the critical minority class events, such as instances of SIB. Our approach, which combines physiological biosignals with detailed movement analysis, seeks to mitigate these challenges by enhancing the sensitivity and specificity of SIB detection in the context of ASD.

The methodological framework developed in this study has potential applications beyond the realm of ASD research. Our approach to studying and identifying repetitive or SIBs could be adapted for use in other conditions characterized by similar behavioral patterns. For instance, this methodology might prove valuable in studying trichotillomania (hair-pulling disorder) or other body-focused repetitive behaviors. The combination of movement data from accelerometers with physiological measures like EDA and skin temperature could provide valuable insights into the manifestation and triggers of these behaviors across different conditions. However, it is crucial to note that any such application would require careful validation and likely adaptation of our algorithms to account for the specific characteristics of each condition. The movement patterns, physiological responses, and behavioral manifestations may differ significantly between ASD and other disorders. This expansion of our approach to other populations could not only broaden its impact but also provide comparative data that might further illuminate the unique aspects of behavioral patterns in ASD.

### 5.1. Limitations and future directions

We acknowledge the sex imbalance in our study, which predominantly includes male participants. This reflects the higher prevalence of ASD diagnoses among males, as well as the enrollment demographics of our program. While our study was conducted in a naturalistic setting with the existing student population, the limited enrollment of female students results in a classroom that is largely male. This naturalistic approach adds ecological validity to our findings by capturing behaviors in a real-world educational context. However, this sex imbalance may introduce bias, potentially limiting the generalizability of our findings to female students with ASD. To improve the generalizability of our model, future research should aim to include data from female students with ASD. Integrating female participants would allow for the development of models that are more representative of the entire ASD population, thereby enhancing the external validity of our findings.

In this study, the sample size was limited to 10 subjects, which constrained our ability to perform meaningful subgroup analyses, such as comparing algorithm performance between younger and older participants. While it is possible that age-related physiological differences could affect the model’s performance, the current dataset does not provide sufficient statistical power to reliably detect such effects. Any age-based conclusions would likely be misleading, given the small sample size. Therefore, although this analysis is a promising direction for future work, we did not pursue it in this study to avoid potential overinterpretation of the data. Future studies with larger cohorts will allow for a more robust investigation of how factors such as age may influence model performance.

It is important to note that our study focused exclusively on individuals with ASD, reflecting TCFD’s dedicated mission and expertise in supporting and studying this population. TCFD’s specialized resources and deep commitment to individuals with autism enabled our in-depth study of ASD-related behaviors. While this focus allowed for rich, targeted data collection, it meant that our current research setting did not include a control group of neurotypical individuals (those without ASD or other neurodevelopmental differences). The performance of our algorithm on neurotypical individuals thus remains an area for future exploration. Theoretical considerations suggest there could be significant differences in movement patterns and physiological responses between ASD and neurotypical populations, which might affect the algorithm’s performance. These could include variations in baseline activity levels, stress responses, or patterns of repetitive movements that are characteristic of ASD but less common in neurotypical individuals. Future research could expand on our findings by including neurotypical participants, potentially broadening the scope of our study while building upon the valuable insights gained from TCFD’s specialized environment. This would help establish the specificity of our approach to ASD-related behaviors and determine the algorithm’s ability to discriminate between ASD-specific behaviors and typical variations in movement and physiological responses. Such research would be crucial in validating the broader applicability and specificity of our method.

Another limitation of our study is the use of a single annotator for ground truth labeling. While this ensures consistency in the annotations, it also introduces potential bias, as the perspective and interpretation of challenging behaviors are limited to one individual. In future studies, involving multiple annotators or utilizing consensus-based annotation methods could enhance the reliability and robustness of the labels.

The study focuses on a broad spectrum of challenging behaviors, operationalized through fifteen distinct actions, such as out-of-seat, elopement, motor stereotypies, aggression, SIB, and others. These behaviors can manifest in varied ways across different physiological and movement signals, adding complexity to their interpretation. While this broad approach allows for the capture of a wide range of behaviors, it may also dilute the specificity of our findings, as each behavior can have different physiological and movement signatures. Future work should consider refining these definitions to better capture the nuances of specific challenging behaviors.

Exploring the integration of video analysis into our research framework presents a promising avenue for future work. Although not within the current study’s scope, video analysis could significantly augment our predictive models by providing rich, contextual insights into behavioral manifestations. Given that our ground truth labels are primarily derived from video data, it is logical to further leverage video analysis to identify challenging behaviors. This modality has the potential to capture detailed interactions and subtle behavioral cues that wearable device data alone may not reveal, thereby enriching the model’s predictive capability.

## 6. Conclusions

This study pioneers a novel approach to detecting challenging behavior in children with ASD in naturalistic settings, integrating physiological biosignals and three-dimensional movement data. Our findings illuminate the complexities of implementing wearable technology and machine learning algorithms beyond controlled environments, revealing complex dynamics of ASD-related behaviors.

Our work emphasizes the importance of developing adaptable, multimodal approaches capable of navigating the inherent variability of daily life. While the performance in detecting challenging behaviors in natural settings leaves room for improvement, this study significantly advances our understanding of the practical application of ASD research. This investigation is a critical step toward bridging the gap between theoretical models and their real-world implementation. It paves the way for more sophisticated, personalized interventions that could markedly improve outcomes for individuals with ASD and their families.

Moving forward, our findings will inform future studies, driving the development of innovative solutions that are both scientifically robust and practically viable in everyday environments. This research reinforces our commitment to improving the management of challenging behaviors in ASD through evidence-based, applicable methodologies.

## Data Availability

The data cannot be made publicly available upon publication because they are owned by a third party and the terms of use prevent public distribution. The data that support the findings of this study are available upon reasonable request from the authors.
